# *In vitro* characterization of probiotic properties of isolated and grown co-cultures on halophyte-based substrate

**DOI:** 10.1007/s10123-026-00786-4

**Published:** 2026-03-09

**Authors:** Stanislav Rudnyckyj, Olha Zhytniakivska, Mette Hedegaard Thomsen, Tjaša Čukajne, Bojana Bogovič Matijašić, Petra Mohar Lorbeg

**Affiliations:** 1 Department of Energy, Aalborg University, Niels Bohrs Vej 8, Esbjerg, 6700 Denmark; 2 Department of Chemistry and Bioscience, Aalborg University, Niels Bohrs Vej 8, Esbjerg, 6700 Denmark; 3 Biotechnical Faculty, Institute of Dairy Science and Probiotics, University in Ljubljana, Domžale, Slovenia

**Keywords:** Potential probiotics, Yeast, Lactic acid bacteria, Bioprospecting, Halophytes

## Abstract

Probiotics are increasingly recognized for their health-promoting effects, yet their performance in unconventional fermentation systems such as halophyte-based substrates remains poorly understood. Halophytes, salt-tolerant plants rich in phenolics and other bioactive compounds, impose selective pressures that may favor robust and stress-tolerant microorganisms. In this study, we assessed the probiotic potential of selected lactic acid bacteria and yeast strains, including *Lactococcus (L.) lactis*, *Bacillus (B.) coagulans* ATCC 7050, *Saccharomyces (S.) cerevisiae*, *Kluyveromyces (K.) marxianus* DSM 7238, and *Cyberlindnera (C.) jadinii* DSM 2361 and DSM 70,163, cultivated on halophyte hydrolysates. In addition, newly isolated strains from halophyte biomass, *Lactiplantibacillus (Lpl.) plantarum*, *Levilactobacillus (Lev.) brevis*, and *Pichia (P.) fermentans*, were bioprospected for their probiotic potential and robustness against relevant environmental stressors. Strains were assessed for viability, tolerance to NaCl and phenol, survival under simulated gastrointestinal conditions, cell surface properties (hydrophobicity, autoaggregation, biofilm formation), antioxidant activity, and antimicrobial effects. Results revealed great variability among tested strains. *Lpl. plantarum* and *L. lactis* demonstrated high salt (> 70% survivability at 5% NaCl) and phenol tolerance (> 80% survivability at 0.3% phenol), strong survivability in simulated digestion (up to ~ 30% survival for *Lpl. plantarum* and < 10% for *L. lactis*), and notable antimicrobial activity against up to three tested pathogens, as well as antioxidant activity (up to ~ 55% DPPH scavenging). *K. marxianus* exhibited high stress tolerance, enhanced antioxidant capacity (up to ~ 70% DPPH scavenging), high survivability after simulated digestion (> 100%), and inhibition of several pathogens. Conversely, *B. coagulans* showed broad antibiotic resistance, low gastrointestinal survivability (< 5%), and poor growth in halophyte-based media. Overall, *Lpl. plantarum* and *K. marxianus* emerged as the most promising candidates for probiotic development and biotechnological applications. These findings highlight halophyte biomass as a selective substrate that favors resilient strains with desirable probiotic traits, supporting its potential in developing sustainable, plant-based fermentation systems.

**Graphical abstract:**

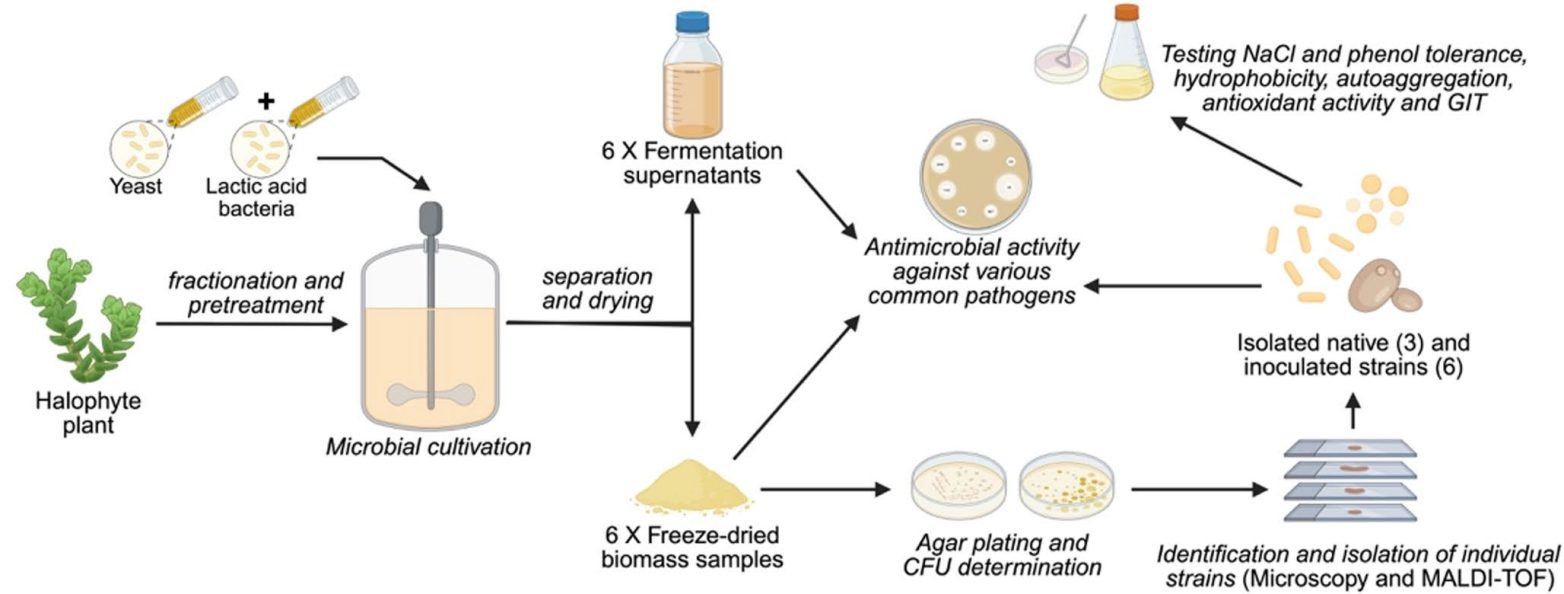

## Highlights

Lpl. plantarum and L. lactis showed robustness and bioactive properties.

K. marxianus exhibited high stress tolerance and probiotic potential.

B. coagulans ATCC 7050 appears least promising probiotic.

## Introduction

Probiotics are increasingly recognized for their health-promoting capabilities, including modulation of the gut microbiota, enhancement of host immunity, and production of bioactive metabolites (Hill et al. 2014; Salminen et al. 1999). This is reflected in the growing global probiotic market, valued at ~ 73 billion USD and projected to reach 85 billion USD by 2027 (Shahbandeh 2025). Rising consumer demand for health-oriented and “natural” products continues to drive interest in functional foods and fermentation-based probiotics (Cosme et al. 2022; Nguyen et al. 2020).

Among probiotic microorganisms, lactic acid bacteria (LAB) and generally recognized as safe (GRAS) yeasts have been extensively studied, particularly in the context of probiotic fermentation and functional foods (Czerucka et al. 2007; Hugot et al. 2023; Khushboo, Karnwal & Malik, 2023; Klein et al. 1998; Maftei et al. 2024; Vergara et al. 2023). Traditionally, plant-based substrates such as cereals (Andrade and de Castro 2023; Ashaolu et al. 2025), legumes (Cichońska and Ziarno 2021), and vegetables (Bernal-Castro et al. 2023) have been explored for probiotic growth. However, these substrates often lack the environmental stressors (salinity, phenolic compounds) that better reflect the conditions probiotics encounter during digestion, limiting the discovery of highly stress-tolerant strains.

Halophyte plants, salt-tolerant species adapted to extreme environments, remain underexplored despite their richness in bioactive compounds, including phenolics, flavonoids, and terpenoids (Hulkko et al. 2022b, 2023a). In addition to their nutritional value, halophytes are sustainable crops, requiring minimal freshwater and thriving in marginal lands, making them an attractive alternative feedstock for functional fermentations (Accogli et al. 2023; Ahmadi et al. 2022; Aziz and Mujeeb 2022; Yadav et al. 2018). Recent studies have highlighted halophyte biomass as a promising resource for biorefinery and fermentation applications due to its abundance, resilience, and unique chemical composition (Fredsgaard et al. 2023; Hulkko et al. 2023b; Lopes et al. 2023). The high salt and phenolic content of halophytes can act as selective pressures, favoring robust and stress-tolerant microorganisms (Rudnyckyj and Thomsen 2025). However, there is limited research on how LAB and yeasts perform in halophyte-based media, or whether co-cultivation can enhance their survival and functional traits. Prior studies have shown that halophyte biomass can support the growth of LAB and yeasts (Alassali et al. 2017; Cybulska et al. 2014; Hulkko 2023; Maoloni et al. 2022), indicating its suitability as a fermentation substrate for these microorganisms.

In particular, probiotic yeasts such as *Saccharomyces cerevisiae*, *Kluyveromyces marxianus*, and *Candida jadinii* have attracted growing interest due to their stress tolerance, bioactive compound production, and safety for human consumption (Fu et al. 2023; Hugot et al. 2023; Moradi et al. 2018). Yet, their potential in halophyte-based fermentation systems has not been systematically evaluated. Similarly, well-known bacterial probiotics such as *Lactococcus lactis* and *Bacillus coagulans* may exhibit distinct physiological responses when cultivated in such challenging substrates (Amoah et al. 2019; Kimoto-Nira et al. 2010; Madana and Sathiavelu 2024; Wang et al. 2023; Yan et al. 2025). However, their potential in such systems has not been systematically evaluated, representing a key knowledge gap this study seeks to address.

Therefore, the first aim of this study was to investigate the performance of selected LAB and yeast strains in halophyte-based media as pre-selected mixed cultures. Followingly, the chosen strains including *L. lactis*, *B. coagulans* ATCC 7050, *S. cerevisiae*, *K. marxianus* DSM 7238, *C. jadinii* DSM 2361, *C. jadinii* DSM 70,163, as well as halophyte-derived isolates *Levilactobacillus brevis* IM 2436, *Lactiplantibacillus plantarum* IM 2435, and *Pichia fermentans* IM 2437 were bioprospected for their robustness to stressors typical of salt-rich plant media and their probiotic potential. More specifically, we evaluated their viability, tolerance to salt and phenolic stress, survival under simulated gastrointestinal conditions, cell surface properties (hydrophobicity, autoaggregation, and biofilm formation), antioxidant activity, and antimicrobial potential. By integrating these assessments, our goal was to identify the most promising strains not only for probiotic development but also for sustainable biotechnological applications in halophyte-based fermentation systems.

The novelty of this study lies in the use of halophyte-derived biomass as a selective, non-sterile fermentation substrate to simultaneously evaluate the performance of inoculated and indigenous microorganisms. Unlike conventional probiotic screening approaches that rely on sterile media and isolated strain testing, this work integrates ecological selection with functional probiotic characterization, enabling identification of robust strains capable of persisting and expressing desirable traits under competitive, plant-based fermentation conditions. This approach provides new insights into the potential of halophyte biomass as a sustainable platform for probiotic and biotechnological applications.

## Materials and methods

### Fermentation of halophyte-based media

The halophyte biomass used for media preparation and isolation of indigenous strains originated from *Salicornia ramosissima* (Riasearch, Portugal), and the cultivation conditions for halophyte-based fermentations had been previously optimized as described in Rudnyckyj et al. (Rudnyckyj et al. 2025a). The halophyte-based media was fermented using combination of bacteria and yeast. Table 1 demonstrates starter cultures used for inoculation in halophyte-based media fermentation. For inoculum preparation, the bacterial cultures were cultivated in MRS broth and incubated for 24 h at 30 °C, except for *B. coagulans* ATCC 7050, which was grown at 37 °C. Yeasts were cultivated in YPD broth for 24 h at 30 °C and 150 RPM, except for *K. marxianus* DSM 7238, which were grown at 37 °C.

**Table 1:** Starter cultures grown on halophyte-based substrate

Code name	Bacterial culture	Yeast culture	Temperature (℃)	Cultivation
LLSC	*Lactococcus lactis* iso.	S. cerevisiae*	32	co-culture
LLSC2	*Lactococcus lactis* iso.	S. cerevisiae*	32	sequential
BCKM	*B. coagulans* ATCC 7050	*K. marxianus* DSM 7238	37	co-culture
BCCJ	*B. coagulans* ATCC 7050	*C. jadinii* DSM 2361	32	co-culture
BCCJ2	*B. coagulans* ATCC 7050	*C. jadinii* DSM 70,163	32	co-culture
BCSC	*B. coagulans* ATCC 7050	S. cerevisiae*	32	co-culture

* commercial dry yeast, Malteserkors tørgær, De Danske Spritfabrikker A/S, Denmark

For bacterial and yeast co-cultures on halophyte-based hydrolysate, 180 mL of hydrolysate slurry was inoculated with 10 mL of yeast inoculum and 10 mL of bacterial inoculum. For sequential cultivation, the yeast inoculum was introduced after an initial 48 h of bacterial growth on the hydrolysate. Sequential cultivation was investigated to evaluate whether an initial anaerobic fermentation phase could improve the growth performance of *L. lactis*, which is less oxygen-tolerant than the other bacterial strains used. Following this initial phase, yeast was introduced and cultivation was continued under aerobic conditions for 24 h.

Cultures were incubated at 32 °C and 150 RPM for a total of 72 h, with exception of *K. marxianus* DSM 7238 with incubation at 37 °C. Fermentation was performed under non-sterile conditions. The samples were centrifuged (SL 16 Centrifuge, Thermo Fisher Scientific, USA) at 3000 RPM for 10 min. Biomass pellets were washed twice with 100 mL of Milli-Q water. To each sample 30 mL of 20% (w/w) sucrose solution was added as cryoprotectant. The washed pellets were stored at − 80 °C overnight, then freeze-dried for two days, and subsequently used for analysis.

### Determination of viable counts (CFU/g) and identification of selected colonies

For CFU quantification by plate counting, 0.10 g of ground freeze-dried co-culture product was mixed with 9.9 mL of sterile ¼ strength Ringer solution (Merck, Germany) and vortexed. After serial dilutions in Ringer solution, samples were spread on M17 (for *L. lactis*), MRS (for *B. coagulans*) and YGC agar (for yeasts), depending on the microorganism used as inoculum. Plates were incubated for 72 h at 30 °C in aerobic conditions, except for MRS plates that were incubated at 37 °C.

After incubation, colonies typical for inoculated microorganisms were counted. Since some of the grown colonies were not typical, they were sent for identification using matrix-assisted laser desorption ionisation time-of-flight mass spectrometry (MALDI-TOF MS), as described in previous work (Mohar Lorbeg et al. 2021). After identification, they were purified by spreading them onto fresh agar plates and cultivated in suitable liquid media.

### Cultivation conditions of strains tested

Six microbial strains used as inoculum and newly isolated strains *Lev. brevis* IM 2436, *Lpl. plantarum* IM 2435, as well as *Pichia fermentans* IM 2437 were further tested. Cultivation of the strains was as follows: tested strains were inoculated (1%) in suitable liquid media and cultivated overnight: *Lpl. plantarum*, *L. lactis* and *Lev. brevis* in MRS broth (Merck, Germany) at 30 °C; *B. coagulans* in MRS broth at 37 °C, *K. marxianus* in SD (VWR, USA) broth at 37 °C; *C. jadinii*, *S. cerevisiae* and *P. fermentans* in SD broth at 30 °C.

### Antimicrobial activity

Antimicrobial activity of tested strains was evaluated against 25 indicator strains, comprising 18 common microbial pathogens and seven fish-specific (Table 2), included due to the increasing application of probiotics in aquaculture and the relevance of halophyte-based substrates for sustainable aquafeed systems.

**Table 2:** Indicator strains used in the present work, hosts infected by pathogen, and their growth temperature

Pathogenic strain	Pathogenic towards	Growth media and temperature (℃)
*Escherichia coli* ATCC 25,922	Mammalians	BHI, 37
*Streptococcus salivarius* M84-01	Mammalians	BHI, 37
*Klebsiella oxytoca* IM 878	Mammalians	BHI, 37
*Staphylococcus aureus subsp. aureus Rosenbach* 1884AL, LMG 10,147	Mammalians	BHI, 37
*Staphylococcus lugdunensis* M33-01	Mammalians	BHI, 37
*Escherichia coli* O8 K88 + ENT- (A)	Mammalians	BHI, 37
*Listeria monocytogenes* ŽM 46 P	Mammalians	BHI, 37
*Salmonella enterica subsp. enterica serovar Enteritidis* IM 186	Mammalians	BHI, 37
*Salmonella enterica subsp. enterica serovar Brandenburg* IM 187	Mammalians	BHI, 37
*Escherichia coli* O157:H7	Mammalians	BHI, 37
*Lactococcus garviae* IM 2397	Aquaculture	BHI, 30
*Aeromonas hydrophila* IM 2392	Aquaculture	BHI, 30
*Aeromonas sobria* IM 2398	Aquaculture	BHI, 30
*Yersinia ruckeri* IM 2395	Aquaculture	BHI, 30
*Serratia marcescens* IM 2396	Aquaculture	BHI, 30
*Aeromonas salmonicida* IM 2393	Aquaculture	BHI, 25
*Aeromonas salmonicida* IM 2394	Aquaculture	BHI, 25
*Staphylococcus epidermidis* LMG 10,474	Mammalians	BHI, 37
*Enterococcus faecalis* LMG 7937	Mammalians	BHI, 37
*Staphylococcus epidermidis* LMG 10,273	Mammalians	BHI, 37
*Listeria monocytogenes* LMG 13,305	Mammalians	BHI, 37
*Salmonella enterica serovar Typhimurium* ATCC 14,028	Mammalians	BHI, 37
*Clostridioides difficile* MW1	Mammalians	RCM, 37 (anaerobic)
*Clostridium perfringens* 4D 485	Mammalians	RCM, 37 (anaerobic)
*Clostridium tyrobutyricum* DSM 2637	Mammalians	RCM, 37 (anaerobic)

For the analysis, freeze-dried biomass samples were prepared as described in paragraph 2.2. while tested strains were cultivated overnight in suitable liquid media. A 5 µL aliquot of prepared biomass sample or strain culture was inoculated on nutrient agar (NA) with the following composition: 1 g of meat extract/L, 2 g of yeast extract/L, 5 g of peptone from meat/L, 5 g of NaCl/L, and 10 g of agar/L. Plates were incubated overnight at 30℃. After incubation, the resulting microbial spots were overlaid with 4 ml of suitable soft agar inoculated with indicator strain (1% v/v). Soft agar was prepared from BHI (Brain Heart Infusion, Merck, Germany) of RCM (Robertson’s Cooked Meat, BioLife, Italy) broth with addition of agar-agar 5 g/L. Prepared plates were incubated under conditions appropriate for indicator strain (Table 2). After incubation, the presence of a clear zone surrounding the microbial colonies indicated antimicrobial activity.

For testing antimicrobial activity of cell free supernatants (CFS) from strains that exhibited preliminary antimicrobial activity, first the soft agar inoculated with indicator strain was poured over NA agar. Subsequently, 5 µL of each sample was applied to designated spot and allowed to dry for 10 min. This process was repeated twice. CFS were obtained from overnight cultures by centrifugation at 3600 ⋅ g followed by filtration of the supernatants through 0.2 μm filters under aseptic conditions. Additionally, the antimicrobial activity of neutralized supernatants (pH adjusted to 7) was evaluated to distinguish between peptide-based and acid-based antimicrobial effects. After incubation, representative inhibition zones were recorded as positive result.

### Antibiotic susceptibility

Minimum inhibitory concentrations (MICs) of 23 antimicrobials were determined by broth microdilution method in accordance with the standard ISO 10,932 (ISO 10932 2010). The antimicrobials used include: ampicillin (AMP), cefoxitin (FOX), chloramphenicol (CHL), ciprofloxacin (CIP), clindamycin (CLI), daptomycin (DAP), erythromycin (ERY), fusidic acid (FUS), gentamicin (GEN), kanamycin (KAN), linezolid (LZD), mupirocin (MUP), penicillin (PEN), quinupristin/dalfopristin (SYN), rifampin (RIF), streptomycin (STR), sulfamethoxazole (SMX), teicoplanin (TEI), tetracycline (TET), tigecycline (TGC), tiamulin (TIA), trimethoprim (TMP), and vancomycin (VAN).

Bacterial strains were propagated on MRS (*Lpl. plantarum*,* Lev. brevis* and *B. coagulans*) or M17 agar (*L. lactis*) and grown colonies were suspended into a sterile ¼ Ringer solution (Merck) until the turbidity matched McFarland standard 1. The bacterial suspensions were then diluted 1000-fold in LSM broth (pH 6.7) consisting of 90% Iso-Sensitest (Oxoid, Basingstoke, England) and 10% MRS broth (Merck), while for *L. lactis* strain Iso-Sensitest broth (pH 7.4) was used. Aliquots of prepared suspensions (50 µl) were distributed into the wells of pre-coated Sensititre™ EUST2 and Sensititre™ EUVENC microtiter plates (Thermo Scientific, UK). Plates were incubated for 48 h at 30 °C in under anaerobic conditions (*Lpl. plantarum* and *Lev. brevis*) or aerobic conditions (*L. lactis*) while plates with *B. coagulans* were incubated aerobically at 37 °C. MIC for each antibiotic tested was determined visually as the lowest concentration at which there is no visible growth was observed. Microbiological cut-off values were adopted by (EFSA 2008).

### In vitro digestion

In vitro digestion was performed following the standardized Infogest protocol (Brodkorb et al. 2019) with minor modifications. Simulated salivary fluid (SSF), simulated gastric fluid (SGF) and simulated intestinal fluids (SIF) were prepared according to Infogest protocol using the following reagents: KCl (Sigma-Aldrich, USA), KH_2_PO_4_ (Merck, Germany), NaHCO_3_ (Merck, Germany), NaCl (Merck, Germany), MgCl_2_·6H_2_O (Sigma-Aldrich, USA), (NH_4_)_2_CO_3_ (Honneywell, USA) and CaCl_2_·2H_2_O (Sigma-Aldrich, USA). The enzymes used were α-amylase from human saliva (Sigma A1031, USA), pepsin and gastric lipase were added as rabbit gastric extract by Lipolytech, France (RGE), pancreatin from porcine pancreas (Sigma P1750, 4xUSP, USA), and bovine bile (Sigma-Aldrich B3883, USA).

Overnight cultures of the tested strains were centrifuged at 3600 ⋅ g for 5 min, and the resulting pellets were washed with 5 ml of phosphate-buffered saline (PBS, pH 7.4). After a second centrifugation, the pellets were resuspended in 1.5 mL SSF. To determine viable count of tested strains prior to simulated digestion, 0.1 mL of cell suspension was serially diluted in ¼ strength Ringer’s solution (Merck) and inoculated into MRS (Merck) or SD (VWR) agar and incubated 72 h under previously described.

For the gastric phase, 1 mL of microbial cell suspension in SSF was mixed with 2.25 mL SGF, 0.48 mL of a solution containing lipase (750 U/mL) and pepsin (25,000 U/mL) (both provided as rabbit gastric extract by Lipolytech), 0.06 mL of 0.1 M HCl, 1.5 µL of 0.3 M CaCl_2_·2H_2_O. The pH was adjusted to 3.0 ± 0.1 and water was added to reach a final volume of 4 mL. The mixture was incubated for two hours at 37 °C with continuous stirring at 75 rpm.

For the intestinal phase, the gastric mixture was supplemented with 2.2 ml of SIF, 1 ml of porcine pancreatin solution in SIF (8 mg/mL of 4xUSP pancreatin, Sigma-Aldrich, USA), 0.5 mL of 160 mM bile salt solution (bovine bile, Sigma-Aldrich, USA) in water, 8 µL of 0.3 M CaCl_2_·2H_2_O, and 30 µL of 1 M NaOH was added to gastric mixture. The pH was adjusted to 7.0 ± 0.1, and distilled water was added to reach a final volume of 8 mL. After incubation at 37 °C for 2 h with gentle agitation (75 rpm), 1 mL of the resulting mixture was used to quantify viable bacteria, as previously described. The analyses were performed in two biological repetitions.

### NaCl and phenol tolerance

For NaCl and phenol tolerance testing of individual strains, MRS medium was used for bacteria and SD medium for yeast, with added salt at final concentrations of 2.5, 5, 7.5, and 10% (w/v) and phenol at 0.1% and 0.3% (w/v). NaCl was added before autoclaving, while phenol was added under sterile conditions after autoclaving. One-day pre-grown microbial cultures were used to inoculate both control and spiked media at an inoculation size of 1% (v/v). Microbial strains were incubated for 24 h at 30 °C, except for *B. coagulans* and *K. marxianus DSM 7238*, which were grown at 37 °C. The overall method followed the procedure described by Madana and Sathiavelu (2024), with the exception of absorbance measurement, which was performed at 630 nm. The results were expressed as growth percentage (% of survivability), and the experiment was performed in biological triplicates. Survivability was calculated relative to control cultures grown under identical conditions without added stressors, which were defined as 100% of survivability.

### Cell surface hydrophobicity and autoaggregation

The assessment of cell surface hydrophobicity and autoaggregation was performed following the protocol described by Jayaram et al. (2025), with minor modifications. LAB strains were cultured in MRS broth at 37 °C, while *L. lactis* was grown at 30 °C. Yeast strains were cultivated in YPD medium at 30 °C, and *K. marxianus* was incubated at 37 °C. After incubation, cultures were centrifuged at 3000 RPM for 10 min, and the resulting cell pellets were washed twice with phosphate-buffered saline (PBS). The optical density OD_600_ of each washed suspension was adjusted to 0.4–0.5.

For the hydrophobicity assay, 1 mL each of xylene, toluene, and hexadecane was added to separate sterile test tubes. Then, 3 mL of microbial suspension was added to each tube. The mixtures were vortexed for 2 min and left undisturbed for 1 h to allow phase separation. Afterward, the aqueous phase was carefully removed, and its OD_600_ was measured to determine hydrophobicity.

For the autoaggregation assay, 10 mL of microbial suspension (OD_600_: 0.4–0.5) was transferred to sterile test tubes. The initial OD_600_ was measured (0 h), and the tubes were incubated at 37 °C. OD_600_ readings were subsequently taken at 2, 4, 8, and 24 h. The autoaggregation and hydrophobicity was calculated as described by (Jayaram et al. 2025). All experiments were conducted in biological triplicates, and results were expressed as percentages for both cell surface hydrophobicity and autoaggregation.

### Biofilm formation

The protocol for staining with crystal violet was adopted from (Kurinčič et al. 2016). After washing twice, the plates were dried with a fan for 15 min. Crystal violet (0.1%, Merck, Germany) was added to each well (100 µl) and incubated for 15 min at room temperature. After staining, the plates were washed and dried as described. The bound crystal violet was dissolved in 100 µl of 96% ethanol (Merck, USA) and the absorbance was measured at 568 nm (Tecan, Switzerland). The measurements were repeated for each strain on three different days with three biological and three technical replicates. The ability of the strains to form biofilms was categorised according to the classification described in Stepanović et al. (2000).

### Antioxidant activity

The antioxidant activity of the cell-free culture supernatant (CFS) and pellet cells obtained from each bacterial and yeast strain was evaluated using the 2,2-diphenyl-1-picrylhydrazyl (DPPH) radical scavenging assay, following the procedures described by Shi et al. (2019) and Kim et al. (2020) with some modification. The overnight cultures of tested strains were centrifuged at 4000 RPM for 10 min. The resulting cell-free culture supernatants were collected and filtered using a 0.22 μm nylon syringe filter (25 mm, Merck, Germany). The cell pellets were washed twice and resuspended in PBS. The optical density (OD_600_) of the resuspended cells was adjusted to 0.5.

To assess the antioxidant activity of the cell-free supernatant, 1 mL of a freshly prepared 0.2 mM DPPH solution in methanol was mixed thoroughly with 0.8 mL of the CFS and incubated in the dark for 30 min. For the assay of the resuspended cell pellets, 0.2 mM DPPH solution and the sample were mixed at a 1:3 (v/v) ratio and incubated under the same conditions. After incubation, the absorbance of the samples was measured at 517 nm. The DPPH radical scavenging activity was calculated using the following formula:

DPPH scavenging activity (%) = (A_blank_ – A_sample_/A_blank_) × 100% (1).

A_sample_ – absorbance of the mixture with CFS or the resuspended cells; A_blank_ – blank sample, the CFS or resuspended cells was replaced with pure MRS broth, YPD medium or the PBS buffer.

### Statistical methods

All trials were performed as triplicates, and the samples were prepared in random order. All results are given as mean values with standard deviation. The analysis of variance (ANOVA) test was used for the analysis of experimental results with single or multiple independent variables. The significance level was set to 0.05 for all performed statistical tests. When necessary, ANOVA was supplemented by Tukey’s Honest Significant Difference (HSD) test. The correlation analysis was performed to investigate linear association between all factors. Pearson’s correlation coefficient (r) was used to quantify these associations, with (r) ≥ 0.7 indicating strong correlation between variables. R version 4.5.1 (Core Team 2020) with RStudio software (v. 2025.05.1 Build 513) was used for all statistical analyses.

## Results and discussion

### Viable counts (Log CFU/g) and strains identification

**Fig. 1:**
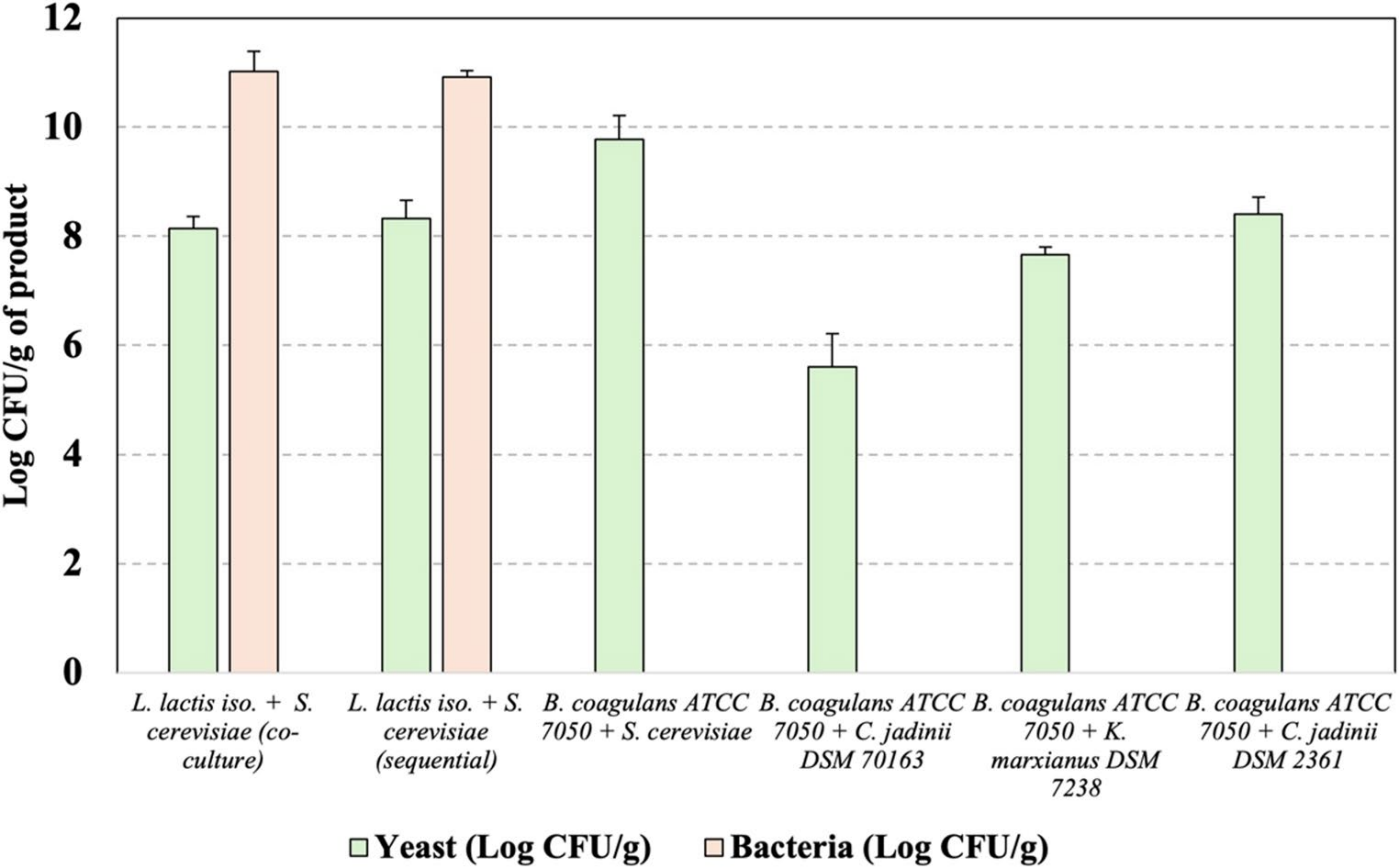
Viable counts (Log CFU/g) of yeast and bacteria in freeze dried biomass grown on halophyte-based media

This section evaluates the ability of inoculated bacterial and yeast strains to grow and persist in halophyte-based media under non-sterile fermentation conditions, as assessed by viable counts and strain identification. Testing of viable counts, as shown in Fig. 1, revealed that bacterial colony formation was observed only in samples inoculated with *L. lactis* and *S. cerevisiae*. Other bacterial–yeast co-cultures listed in Table 3, consisting of *B. coagulans* and selected yeast strains, displayed apparent growth on selective plates for bacteria. However, microscopic analysis confirmed that these were yeast cells. This finding was further validated by MALDI-TOF analysis. Therefore, we can conclude that the halophyte-based hydrolysate did not support effective growth of the *B. coagulans* strain.

**Table 3:** Identified strains in freeze-dried biomass products

Code name	Bacterial inoculum	Yeast inoculum	Identified bacteria	Identified yeast
LLSC	*L. lactis iso.*	*S. cerevisiae*	*L. brevis*,* P. acidilactici*,* L. plantarum*	*S. cerevisiae*
LLSC2	*L. lactis iso.*	*S. cerevisiae*	*L. brevis*,* P. acidilactici*,* L. plantarum*	*S. cerevisiae*
BCSC	*B. coagulans* ATCC 7050	*S. cerevisiae*	N/A	*P. fermentans*,* C. jadinii*
BCCJ	*B. coagulans* ATCC 7050	*C. jadinii* DSM 70,163	N/A	*P. fermentans*
BCKM	*B. coagulans* ATCC 7050	*K. marxianus* DSM 7238	N/A	*K. marxianus*
BCCJ2	*B. coagulans* ATCC 7050	*C. jadinii* DSM 2361	N/A	*P. fermentans* IM 2437, *C. jadinii*

Overall, the results indicate that the highest CFU/g was achieved by the co-culture of *L. lactis* and *S. cerevisiae* regardless of time the yeast was inoculated, indicating that bacterial metabolites do not negatively affect the growth of *S. cerevisiae.* Accordingly, sequential cultivation did not result in improved bacterial or yeast growth compared to co-cultivation, suggesting that this approach had no considerable effect on microbial growth under the conditions tested. Among yeasts, *S. cerevisiae* culture achieved highest CFU/g counts, followed by *C. jadinii* DSM 2361and *K. marxianus*.

**Fig. 2:**
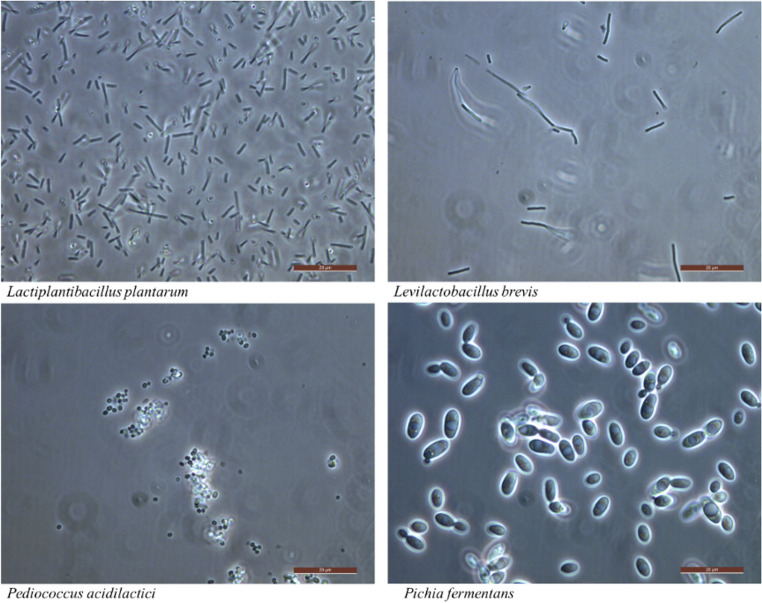
Microscopic images of *Lpl. plantarum.*,* Lev. brevis*,* P. acidilactici*, and the *yeast P. fermentans*

Subsequent investigation of the grown colonies using microscopy, shown in Fig. 2, combined with MALDI-TOF demonstrated that the originally inoculated microorganisms were, in some cases, absent or accompanied by naturally occurring strains from the halophyte-based biomass, as shown in Table 3; Fig. 2.

The observation that naturally occurring microorganisms outcompeted the inoculated strains in several halophyte-based substrates can be attributed to the inherent antimicrobial properties of halophytic plants. Halophytes are known to be rich in polyphenols, flavonoids, alkaloids, and terpenoids, many of which possess strong antibacterial and antifungal activities (Correia et al. 2024; Hulkko et al. 2022a; Ksouri et al. 2012). In addition, their high salt content creates osmotic stress that selectively favors halotolerant or stress-resistant microbes, while inhibiting more sensitive strains (Rudnyckyj and Thomsen 2025). This dual effect of phytochemicals and saline stress likely explains why some inoculated bacteria (e.g. *B. coagulans*) failed to establish detectable populations. Although the superior survivability of indigenous microorganisms under halophyte-based conditions is expected, their dominance alone does not imply probiotic functionality, which requires targeted phenotypic and functional characterization.

Interestingly, the co-inoculation of *L. lactis* and *S. cerevisiae* supported the growth of indigenous lactic acid bacteria (*Lev. brevis*, *P. acidilactici*, and *Lpl. plantarum*). This may be explained by metabolic cross-feeding and protective effects of yeast, which have previously been reported to enhance LAB survival in plant-based fermentations (González-Orozco et al. 2023; Healy et al. 2023; Wang et al. 2023). Yeasts can provide essential growth factors, scavenge oxygen, and modulate redox potential, thereby facilitating bacterial persistence (Ponomarova et al. 2017; Sieuwerts et al. 2018; Vorob’eva et al. 2016). However, in the case of co-culture with *B. coagulans*, no similar synergy was observed, suggesting strain-specific responses to the halophyte matrix. Notably, the same strain, *B. coagulans* ATCC 7050, has previously demonstrated improved growth and nutrient utilization when co-cultured with *C. jadinii* on fermentation wastewater (Liu et al. 2019), highlighting the importance of the cultivation environment in shaping interspecies interactions.

The replacement of inoculated strains by native halophyte-associated microbes highlights the strong selective pressure exerted by this substrate. Previous studies have shown that halophyte extracts inhibit a wide range of bacteria, including foodborne pathogens (*Escherichia coli*, *Staphylococcus aureus*, *Listeria monocytogenes*) (Campana et al. 2022; Correia et al. 2024; Yassien et al. 2021). This aligns with our findings where inoculated strains were suppressed or displaced, while more tolerant species, particularly yeasts, dominated.

Remarkably, the strains of *P. fermentans* are already used in the food industry, valued for its ability to enhance aroma (Gao et al. 2024), modulate acidity (Yi et al. 2025), and contribute to the overall quality of fermented products (Zhong et al. 2021). It has previously been tested and shown to possess probiotic characteristics, owing to its safety profile, antimicrobial activity, and production of valuable fatty acids (de Melo Pereira et al. 2014; Silva et al. 2011).

Taken together, these results underline the importance of substrate-specific ecological interactions in determining microbial community outcomes. From an applied perspective, they suggest that halophyte-based media may act as a selective environment that favors resilient, robust species such as *S. cerevisiae*,* C. jadinii*, *P. fermentans* and present LAB. This has potential implications for developing novel fermented products with unique functional properties, while also raising challenges for strain selection and stability during fermentation.

### Antimicrobial activity

A total of 15 samples were evaluated for antimicrobial activity against 25 pathogenic strains, representing three distinct categories: (1) freeze-dried biomass obtained from halophyte-based co-culture fermentations, reflecting community-level antimicrobial effects; (2) individual bacterial and yeast strains used as inocula, enabling assessment of strain-specific antimicrobial activity; and (3) indigenous isolates recovered from halophyte-based fermentations, allowing evaluation of antimicrobial potential emerging from substrate-driven microbial selection. This categorization was applied to distinguish between antimicrobial effects arising from mixed microbial communities and those attributable to individual strains.

In total, inhibition was observed against six pathogens, as summarized in Table 4. Notably, strains such as *C. jadinii* DSM 70,163, *C. jadinii* DSM 2361, *K. marxianus* DSM 7238, and *L. lactis* demonstrated inhibitory effects against multiple pathogens, indicating their probiotic potential. To confirm the presence of antimicrobial activity, cell free supernatants from individual cultures were tested. The results indicated that *L. lactis* isolate and *Lpl. plantarum* exhibited antimicrobial activity against *Aeromonas hydrophila* IM 2435 (fish isolate). Antimicrobial activity against fish pathogens is particularly relevant for aquaculture applications, where probiotics are increasingly used to suppress pathogenic bacteria, reduce disease outbreaks, and decrease reliance on antibiotics (Elsegeny et al. 2025; Pereira et al. 2022). However, after neutralization of the supernatants, we did not confirm activity of tested CFS against any indicator bacteria. These findings suggest that the observed antimicrobial effects were most likely attributable to the production of organic acids by the tested strains.

**Table 4:** Antimicrobial activity of freeze-dried biomass obtained after co-culture of LAB and yeasts on halophyte-based hydrolysates, individual yeast and bacterial strains used in co-cultures, and isolates from halophyte-based products. Antimicrobial activity was assessed qualitatively based on the presence of growth Inhibition zones. Detection of a strain or freeze-dried biomass under a given indicator organism indicates the presence of inhibitory activity, whereas cells with (-) indicate no detectable Inhibition

Indicator bacteria	Freeze-dried biomass	Individual yeast strains	Individual bacterial strains
*Staphylococcus lugdunensis* M33-01	BCSC, BCKM, and BCCJ2	*C. jadinii* DSM 70,163, *C. jadinii* DSM 2361, and *K. marxianus* DSM 7238	*B. coagulans* ATCC 7050
*Aeromonas hydrophila* (fish isolate)	(-)	(-)	*L. lactis iso. and Lpl. plantarum* IM 2435
*Aeromonas sobria* (fish isolate)	(-)	(-)	*L. lactis iso.*
*Staphylococcus epidermidis* LMG 10,474, ATCC 14,990	(-)	*C. jadinii* DSM 70,163, *C. jadinii* DSM 2361, and *K. marxianus* DSM 7238	*L. lactis iso.*
*Staphylococcus epidermidis* LMG 10,273, ATCC 12,228	(-)	*C. jadinii* DSM 70,163, *C. jadinii* DSM 2361, and *K. marxianus* DSM 7238	*L. lactis iso.*
*Clostridium difficile* MW1	(-)	(-)	*L. lactis iso.*,* L. plantarum* IM 2435, and *Lev. brevis IM 2436*

Antimicrobial activity is a desirable probiotic trait, as it contributes to competitive exclusion of pathogens (Arena et al. 2018), supports microbial balance within the gastrointestinal tract, and may reduce pathogen colonization (Arena et al. 2018; Liévin-Le Moal and Servin 2014). Overall, antimicrobial activity was primarily associated with individual bacterial and yeast strains rather than freeze-dried co-culture biomass, indicating that inhibitory effects were strain-dependent rather than arising from the fermented matrix itself. In particular, LAB, including *L. lactis* iso. and *Lpl. plantarum* IM 2435, exhibited inhibitory activity against both Gram-positive pathogens and fish-associated *Aeromonas spp.*, whereas several co-culture-derived biomass samples showed no detectable inhibition. These findings suggest that antimicrobial potential in halophyte-based systems is largely determined by the functional properties of specific strains rather than by collective fermentation effects.

Notably, *C. jadinii* DSM 70,163, *C. jadinii* DSM 2361, and *K. marxianus* DSM 7238 were effective against multiple *Staphylococcus* strains. This aligns with previous reports indicating that yeasts can secrete organic acids, ethanol, and volatile compounds that suppress bacterial growth (Balabekyan et al. 2018; Georgescu et al. 2024). For instance, fermentation with *C. jadinii* has been shown to enhance antimicrobial activity against multiple pathogens, including *Staphylococcus spp.* (Eom et al. 2013). Similarly, *K. marxianus* has demonstrated the ability to inhibit the growth of various pathogens, also including *Staphylococcus spp.* (Balabekyan et al. 2018; Saber et al. 2017). Moreover, it has been demonstrated that the ability of yeast to co-aggregate with pathogens is a major factor contributing to its antimicrobial activity (Georgescu et al. 2024; Ma et al. 2023; Rossouw et al. 2015). For instance, *S. cerevisiae* strains demonstrated strong co-aggregation with pathogenic *E. coli* and *S. aureus*, significantly contributing to growth inhibition of pathogens (Cong et al. 2025). The detection of antimicrobial activity in *L. lactis*,* Lev. brevis* and *Lpl. plantarum* isolates is consistent with earlier findings that LAB produce bacteriocins (Almeida et al. 2024; Moiseenko et al. 2024), hydrogen peroxide (Ibrahim et al. 2021), and organic acids with broad-spectrum inhibitory activity.

### NaCl and phenol tolerance

To assess the robustness of the tested strains under stress conditions relevant to both halophyte-based fermentation and gastrointestinal survival, NaCl and phenol tolerance assays were performed. NaCl and phenol tolerance are commonly used indicators of a culture’s ability to survive the host’s digestive system, as high osmotic stress and the presence of phenolic compounds result from the breakdown of lignocellulosic fibers during digestion (Madana and Sathiavelu 2024; Reuben et al. 2019). Therefore, strong tolerance to these compounds suggests a higher likelihood of survival in gastrointestinal conditions. Additionally, this analysis is particularly relevant to halophyte-based hydrolysates, which typically contain high concentrations of salt over 5% (w/v) and phenolics over 0.1% (w/v). Evaluating microbial tolerance to these stressors can help identify the most promising strains for further development and prioritization in bioprocessing applications.

**Fig. 3:**
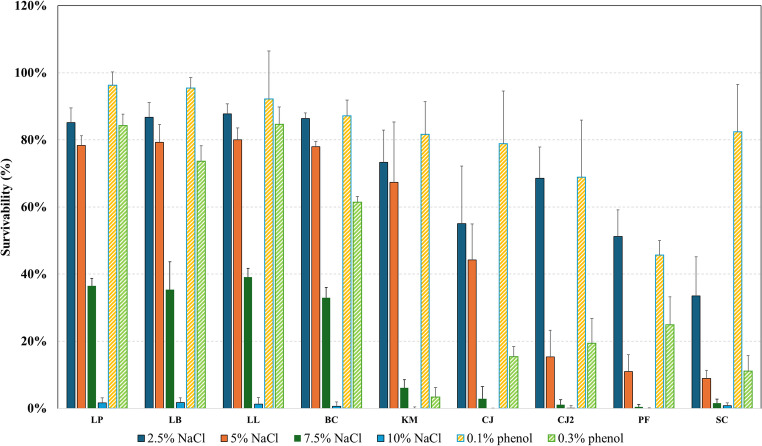
NaCl and phenol tolerance of selected and isolated cultures. LP: *Lpl. plantarum*; LB: *Lev. brevis*; LL: *L. lactis*; BC: *B. coagulans*; KM: *K. marxianus*; CJ: *C. jadinii* DSM 70,163; CJ2: *C. jadinii* DSM 2361; PF: *P. fermentans*; SC: *S. cerevisiae*. Survivability is expressed as a percentage relative to unstressed control cultures, which were defined as 100% survivability

As shown in Fig. 3, bacterial cultures exhibited over 80% survivability at 2.5% NaCl (w/v) and over 70% at 5% NaCl (w/v) compared to the control. However, as salt concentration increased, cell survivability significantly declined, with nearly no growth observed at 10% NaCl. Notably, all bacterial cultures demonstrated robust salt tolerance. These findings align with previous results showing that *L. lactis* performs well in halophyte-based media, and that *Lpl. plantarum* and *Lev. brevis* should be adapted to saline environments due to their halophyte origin. This agrees with earlier studies showing that LAB are inherently salt-tolerant (Rudnyckyj and Thomsen 2025), especially when isolated from salt-rich biomass (Han et al. 2025; Papadopoulou et al. 2023).

Among the yeast cultures, *K. marxianus* showed the highest salt tolerance, maintaining 73% survivability at 2.5% NaCl and 67% at 5% NaCl relative to the control. Overall, yeasts were more sensitive to salt stress, particularly *S. cerevisiae* and the isolated *P. fermentans*, which showed only 34% and 51% survivability at 2.5% NaCl, respectively. This result is somewhat unexpected, as *P. fermentans* originated from halophyte biomass, and *S. cerevisiae* previously demonstrated strong growth in halophyte-based media. However, it is possible that in mixed cultures, different species may support each other in tolerating stressors such as high salinity (Vorob’eva et al. 2016; Yao et al. 2020). In general, yeasts are salt-tolerant, as it was shown that strains of *Pichia* and *Saccharomyces* remain viable even at 10% NaCl (Sengun et al. 2024). However, salt tolerance varies considerably between individual strains of same genus (Alkalbani et al. 2022b).

For phenol tolerance, *Lpl. plantarum*, *Lev. brevis*, *L. lactis*, and *B. coagulans* exhibited strong resistance, maintaining over 85% survivability at 0.1% phenol (w/v). Moreover, *L. plantarum* and *L. lactis* retained more than 80% survivability even at 0.3% phenol (w/v). These findings are consistent with previously reported results showing that *Lpl. plantarum* can maintain high viable cell counts in substrates containing elevated phenol concentrations (0.4% w/v) (Katiku et al. 2022). Based on these results, *Lpl. plantarum*, *Lev. brevis*, and *L. lactis* can be considered promising and robust candidates for fermentation in complex biomass media. For yeasts, the results were less conclusive. *K. marxianus*, *S. cerevisiae*, and *C. jadinii* showed approximately 80% survivability at the same phenol concentration, while *P. fermentans* again exhibited low tolerance, similar to its response to salt stress. Although *P. fermentans* was isolated from halophyte biomass, its low tolerance to salt and phenolic compounds may reflect adaptation to specific micro-niches within the halophyte matrix where stress exposure is reduced. In complex microbial communities, yeasts may benefit from protective effects provided by co-occurring microorganisms or plant-derived structures, allowing survival without requiring high intrinsic stress tolerance (Ferreira et al. 2022; Rudnyckyj et al. 2025b). Furthermore, halophyte-associated microorganisms are not necessarily halophilic, but may instead persist transiently or occupy localized environments with moderated salinity and phenolic concentrations. Similar niche-dependent stress responses have been reported for yeasts isolated from plant-based fermentations (De Vuyst et al. 2014; Fleet 2003). Overall, *K. marxianus* seems as the most promising among tested yeast strains with relatively high salt and phenol tolerance.

### In vitro digestion

**Fig. 4:**
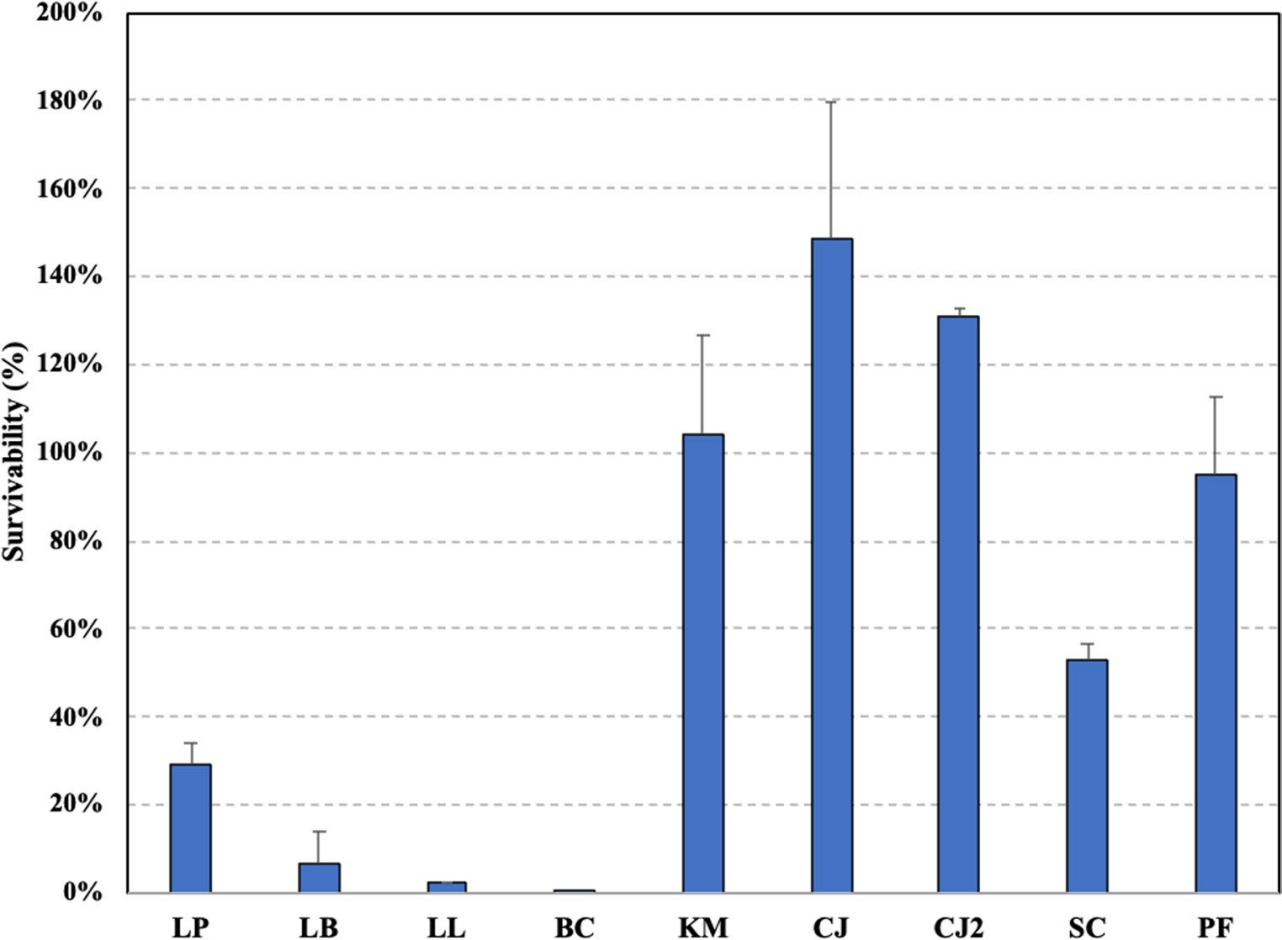
Survival of tested strains after in vitro digestion. LP: *Lpl. plantarum*; LB: *Lev. brevis*; LL: *L. lactis*; BC: *B. coagulans*; KM: *K. marxianus*; CJ: *C. jadinii* DSM 70,163; CJ2: *C. jadinii* DSM 2361; PF: *P. fermentans*; SC: *S. cerevisiae*

The survival of selected bacterial and yeast strains under simulated gastrointestinal conditions was evaluated using an in vitro digestion model to estimate their potential probiotic viability after oral consumption. Based on the results presented in Fig. 4, yeast strains exhibited greater tolerance to in vitro digestion compared to bacterial strains. All yeast cultures, except *S. cerevisiae*, maintained viability even after gastrointestinal tract (GIT) digestion, while number of *S. cerevisiae* decreased (53% survivability). Among bacterial strains, *Lpl. plantarum* demonstrated the highest survivability (30% survival), while passage through in vitro digestion significantly affected viability of *B. coagulans*. Recovery of *L. lactis* and *Lev. brevis* strains after digestion was 2% and 6% respectively.

Similarly, previous studies have reported that yeasts are commonly tolerant to in vitro digestion conditions, showing only a modest reduction in CFU (Alkalbani et al. 2022a). Moreover, systematic reviews have demonstrated that species belonging to the genera *Saccharomyces* and *Pichia* exhibit an average survival rate of approximately 90% following in vitro gastric digestion (Alkalbani et al. 2022b). In the case of LAB strains, similar results were achieved for *Lpl. plantarum* (20–40% survivability) after in vitro digestion, however, it is highly strain dependent (Nagata et al. 2009). Similar results have been reported for other LAB such as *Lev. brevis* (Jurkowski et al. 2019)d *lactis* (Dobson et al. 2011). Conversely, in contrast to our results, a previous study testing the same strain, *B. coagulans* ATCC 7050, demonstrated minimal reduction in CFU following in vitro digestion (Mazzantini et al. 2022). This could be explained by the fact that only intestinal fluid was used for testing of survival, indicating that the strain is highly sensitive to low pH and/or gastric enzymes included in our study. This discrepancy may reflect differences in simulated intestinal fluid protocols or matrix effects, suggesting strain responses are highly context-dependent.

### Cell surface hydrophobicity and autoaggregation

**Fig. 5:**
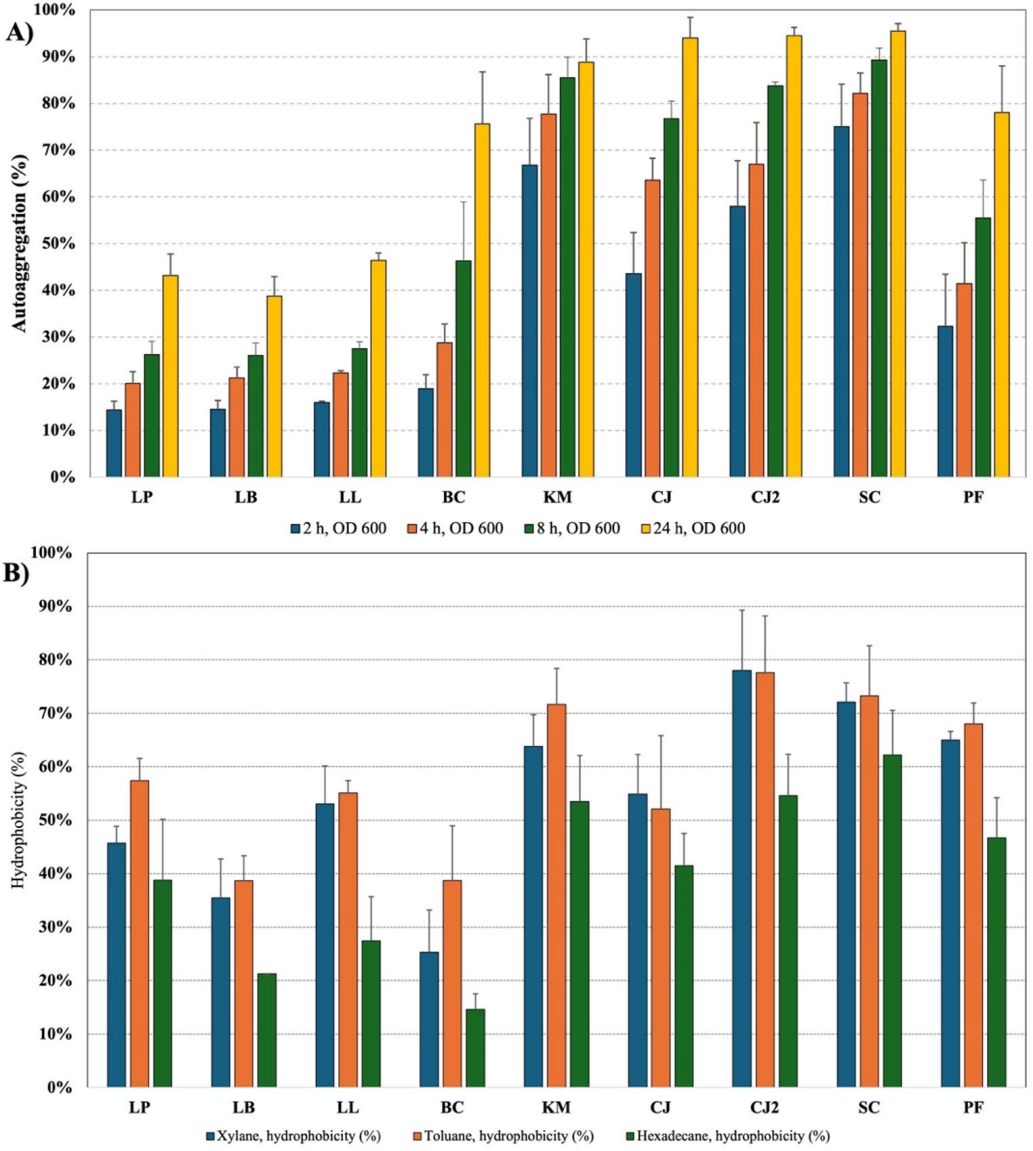
**A**) Autoaggregation and **B**) Cell surface hydrophobicity. LP: *Lpl. plantarum*; LB: *Lev. brevis*; LL: *L. lactis*; BC: *B. coagulans*; KM: *K. marxianus*; CJ: *C. jadinii* DSM 70,163; CJ2: *C. jadinii* DSM 2361; PF: *P. fermentans*; SC: *S. cerevisiae*

Cell surface hydrophobicity and autoaggregation are important probiotic traits, as they are associated with adhesion to intestinal epithelial surfaces and contribute to effective colonization of the host gastrointestinal tract. Moreover, strong autoaggregation can facilitate microbial persistence and enhance competitive exclusion of pathogens by limiting their attachment to host tissues (Gorreja and Walker 2022; Monteagudo-Mera et al. 2019). Autoaggregation, shown in Fig. 5A, increased over time for all strains, with the highest values consistently recorded at 24 h. Yeast strains showed markedly higher aggregation than bacterial strains. By 24 h, *S. cerevisiae*, *C. jadinii*, and *K. marxianus* exceeded 89–95% aggregation, while bacterial strains such as *Lpl. plantarum*, *Lev. brevis*, and *L. lactis* remained below 50%. *B. coagulans* and *P. fermentans* displayed intermediate aggregation (75–78%), higher than other bacteria but lower than most yeasts. A comprehensive assessment of potential probiotic yeasts reported that autoaggregation typically ranges from 60 to 90% in *Saccharomyces*, 30–85% in *Pichia*, 15–90% in *Kluyveromyces*, and 0–99% in *Candida* (*Cyberlindnera*) (Alkalbani et al. 2022b). These findings highlight the substantial natural variability in autoaggregation capacity among yeast strains and confirm that the yeasts investigated in our study fall on the higher end of the autoaggregation spectrum. In the case of LAB, autoaggregation typically ranges between 20 and 50%, as reported for *Lpl. plantarum* (Tuo et al. 2013) d *lactis* (Zhang et al. 2015). Similarly, for *B. coagulans*, values have been shown to vary widely, from as low as 5% to over 70% (Konuray Altun and Erginkaya 2021).

Hydrophobicity profiles, shown in Fig. 5B, varied both by strain and by solvent. Toluene and xylene yielded similarly high hydrophobicity values, while hexadecane produced the lowest in half of the strains. Yeast strains, particularly *C. jadinii* DSM 2361 and *S. cerevisiae*, showed the highest overall hydrophobicity (average > 70% in toluene and xylene). Among bacteria, *Lpl. plantarum* exhibited relatively high hydrophobicity in toluene (~ 60%), while *B. coagulans* showed the lowest across all solvents (< 20% in hexadecane). Similarly, to autoaggregation, a comprehensive review of potential probiotic yeasts showed that hydrophobicity typically ranges from 60 to 90% in *Saccharomyces*, 25–65% in *Pichia*, 30–95% in *Kluyveromyces*, and 25–90% in *Candida* (*Cyberlindnera*) (Alkalbani et al. 2022b). These findings demonstrate the substantial inherent variability in hydrophobicity among yeast strains and confirm that the yeasts examined in our study fall on the higher end of this range. In the case of LAB strains, hydrophobicity typically varies considerably on a strain-to-strain basis (Kaur et al. 2017; Tuo et al. 2013).

To conclude, the data indicates a clear distinction between bacterial and yeast strains in terms of both autoaggregation and hydrophobicity. Yeast strains demonstrated superior autoaggregation capacity and generally higher hydrophobicity compared to bacterial strains, suggesting a greater potential for adhesion to host epithelial surfaces, an important probiotic characteristic. The strong surface properties of yeasts may contribute to better persistence in the gastrointestinal tract, whereas among the bacteria, *B. coagulans* showed the best aggregation potential, while *Lpl. plantarum* combined moderate autoaggregation with relatively high hydrophobicity, making them promising candidates for probiotic applications.

The ANOVA test confirmed that variations in hydrophobicity among strains were statistically significant for all tested solvents. Likewise, for autoaggregation, differences between strains were also statistically significant. Yeast–yeast comparisons generally showed no significant differences, except for *P. fermentans*, which aggregated significantly less than the other yeast strains. Among bacteria, most strains performed similarly, with the exception of *B. coagulans*, which exhibited significantly higher autoaggregation compared to the other bacterial strains.

The results showed a mild correlation between hydrophobicity and autoaggregation, with Pearson’s correlation coefficients (r) ranging from 0.40 to 0.62. Since only values of *r* ≥ 0.7 are considered indicative of a strong correlation, these associations remain moderate. This contrasts with previous studies, which reported no correlation between hydrophobicity and autoaggregation in probiotic bacteria (Tuo et al. 2013; Vlková et al. 2008). However, a relationship between these traits is biologically plausible and has been supported in other studies, where higher hydrophobicity was directly linked to stronger autoaggregation (Aslim et al. 2007; Rahman et al. 2008). This connection is logical, as a more hydrophobic microbial cell surface increases the likelihood of cell–cell interactions, thereby enhancing coagulation (aggregation).

### Biofilm formation

**Fig. 6:**
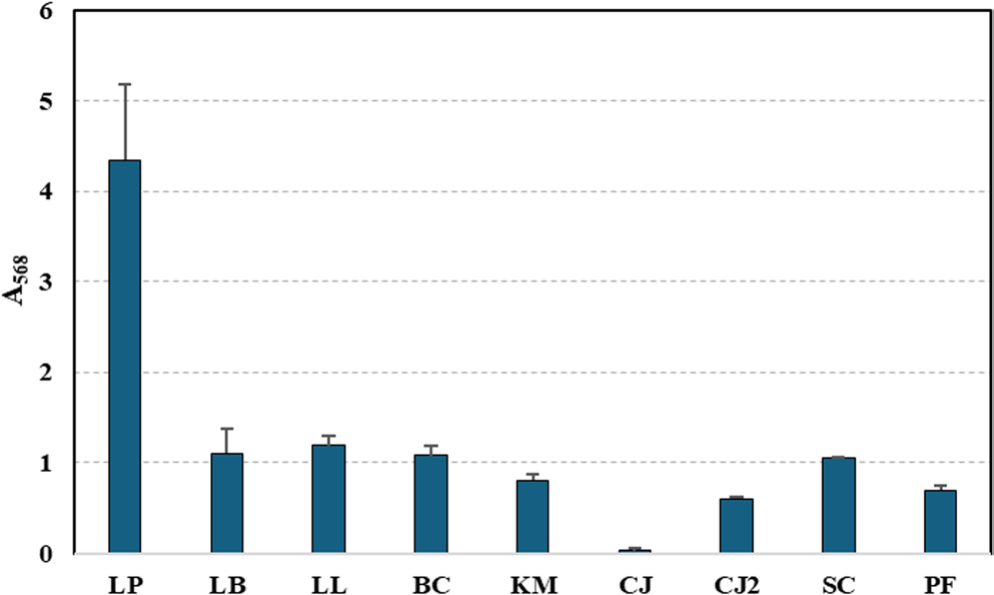
Biofilm formation. LP: *Lpl. plantarum*; LB: *Lev. brevis*; LL: *L. lactis*; BC: *B. coagulans*; KM: *K. marxianus*; CJ: *C. jadinii* DSM 70,163; CJ2: *C. jadinii* DSM 2361; SC: *S. cerevisiae*; PF: *P. fermentans*

Biofilm formation is considered an important probiotic trait, as it can enhance microbial persistence and stability under gastrointestinal conditions. In probiotic strains, biofilm formation may promote prolonged colonization and increase resistance to environmental stresses, thereby supporting sustained functional activity within the host (Salas-Jara et al. 2016; Yao et al. 2025). Biofilm formation, expressed as absorbance at 568 nm after crystal violet staining, varied considerably among the tested strains, as shown in in Fig. 6. *Lpl. plantarum* exhibited the highest biofilm production (A568 ≈ 4.3), significantly surpassing all other cultures. Moderate biofilm formation was observed for *Lev. brevis*, *L. lactis*, *B. coagulans*, and *S. cerevisiae*, with absorbance values around 1.0–1.2. *K. marxianus*, *P. fermentans*, and *C. jadinii* DSM 2361 displayed lower biofilm formation (A568 < 0.8), while *C. jadinii* DSM 70,163 showed negligible biofilm production (A568 ≈ 0.05). ANOVA confirmed that differences in biofilm formation were statistically significant among all strains (*p* < 0.001). However, Tukey’s HSD test indicated that most microbial strains (*Lev. brevis*, *L. lactis*, *B. coagulans*, *K. marxianus*, *S. cerevisiae*, *C. jadinii* DSM 2361, and *P. fermentans*) did not significantly differ from one another (*p* > 0.05). The most pronounced significant difference in biofilm formation was observed in *Lpl. plantarum*, while *C. jadinii* DSM 70,163 showed significantly lower to practically absent biofilm production.

These findings indicate substantial inter-strain variability in biofilm-forming capacity. *Lpl. plantarum* demonstrated a pronounced ability to form biofilms, which could enhance its persistence and colonization potential in complex microbial environments. In contrast, most yeast strains, particularly *C. jadinii* DSM 70,163, exhibited weak biofilm formation, suggesting limited surface adherence. Such differences may have implications for strain selection in probiotic or fermentation applications, especially where biofilm-associated resilience is desirable. Overall, the results are consistent with previously reported findings. For instance, *Lpl. plantarum* is well known for its strong biofilm-forming ability (Carvalho et al. 2021; Rezaei et al. 2021), and LAB in general have been extensively studied for their capacity to produce biofilms. Yeast cultures have also been shown to form biofilms, including *K. marxianus* (Youn et al. 2022)d *cerevisiae* (Speranza et al. 2020). However, there is little direct evidence in the literature regarding biofilm formation by *C. jadinii*. Our results therefore provide novel evidence that *C. jadinii* is capable of forming biofilms, although this ability appears to vary significantly between strains. The observed difference in biofilm-forming capacity between *C. jadinii* DSM 70,163 and DSM 2361 highlights pronounced strain-level variability, which may arise from differences in genetic regulation of cell surface properties or extracellular matrix production.

### Antioxidant capacity

**Fig. 7:**
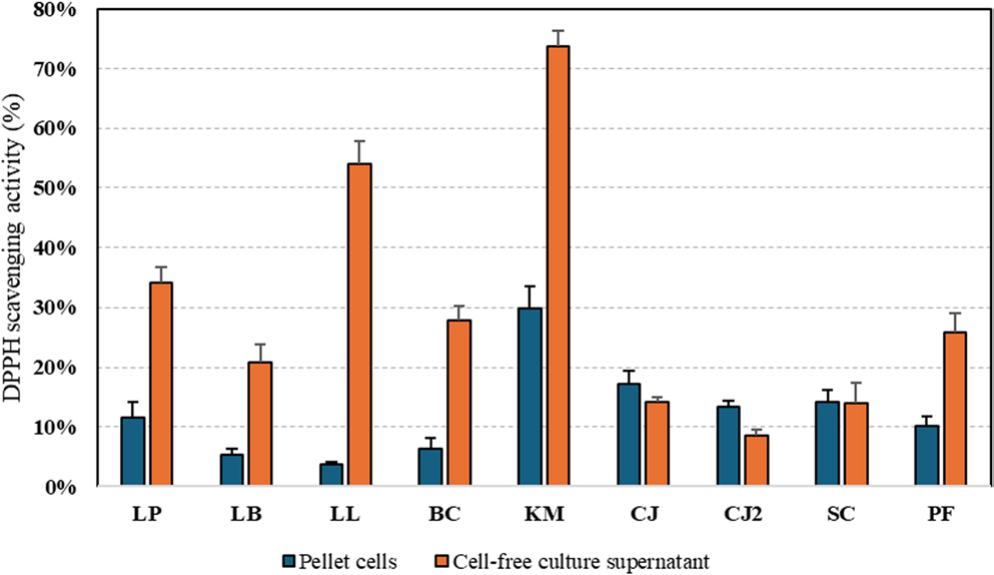
Antioxidant activity based on DPPH scavenging activity (%). LP: *Lpl. plantarum*; LB: *Lev. brevis*; LL: *L. lactis*; BC: *B. coagulans*; KM: *K. marxianus*; CJ: *C. jadinii* DSM 70,163; CJ2: *C. jadinii* DSM 2361; PF: *P. fermentans*; SC: *S. cerevisiae*

Antioxidant activity is a desirable probiotic trait, as it may contribute to the mitigation of oxidative stress in the host gastrointestinal environment. Probiotic strains with antioxidant capacity have been suggested to support intestinal health by reducing reactive oxygen species and protecting host tissues from oxidative damage (Amin et al. 2025; St-Amant and Bergdahl 2023; Wang et al. 2017).The antioxidant capacity, expressed as DPPH radical scavenging activity, varied widely among the tested strains and between pellet cells and cell-free culture supernatants (Fig. 7). For all strains, the cell-free culture supernatant exhibited higher scavenging activity than the corresponding pellet fraction, thus suggesting that compounds with antioxidant activity were predominantly extra-cellular. *K. marxianus* showed the highest antioxidant potential, with supernatant activity exceeding 70% and pellet activity around 30%. *L. lactis* also demonstrated strong supernatant activity of 54%, followed by *Lpl. plantarum* with 34%. Moderate supernatant activities were observed for *P. fermentans*, *B. coagulans*, and *Lev. brevis* (20–30%), while *C. jadinii* DSM 70,163, *S. cerevisiae*, and *C. jadinii* DSM 2361 exhibited the lowest values (< 20%). Pellet fractions showed comparatively low scavenging activities across all strains, generally below 30%, with *K. marxianus* again being the top performer. Overall, the results demonstrate that extracellular metabolites in the culture supernatant exhibited substantially higher antioxidant activity compared to cell-associated components. Among the tested strains, *K. marxianus* showed the highest radical-scavenging capacity, indicating strong potential for postbiotic production. Considering that DPPH radical scavenging values above 50% are generally classified as strong antioxidant activity, and those exceeding 70% as very strong (Kedare and Singh 2011; Silva et al. 2024), both *L. lactis* and *K. marxianus* can be regarded as promising probiotic candidates with notable antioxidant potential. The ANOVA test confirmed that the differences in DPPH radical scavenging activity between pellet cells and cell-free culture supernatants among the investigated strains were statistically significant (*p* < 0.001), with a few exceptions, primarily in the yeast cultures (*C. jadinii* and *S. cerevisiae*), which exhibited comparable antioxidant activity.

Comparable ranges of antioxidant activity, based on DPPH radical scavenging, have previously been reported for LAB, with *Lpl. plantarum* showing 5–40% and *L. lactis* 20–55% activity (Kaur et al. 2017). In contrast, no prior studies have documented such high antioxidant capacity in *K. marxianus*. While *Saccharomyces boulardii* has been shown to reach around 60% in DPPH radical scavenging activity (Menezes et al. 2020) and *Kluyveromyces lactis* in range of 60% to 80% in DPPH radical scavenging activity (Ragavan and Das 2020), research on *K. marxianus* in this context remains limited. Recent findings suggest that the antioxidant properties of *K. marxianus* could be closely linked to exopolysaccharide production (Xu et al. 2025).

### Antibiotic susceptibility

**Fig. 8:**
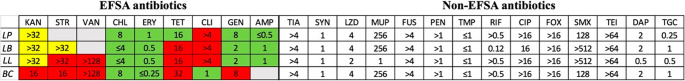
Antimicrobial susceptibility of bacterial strains. For EFSA-listed antibiotics MICs (mg/L) exceeding cut-off values are indicated as red, yellow indicates inconclusive results (the highest concentration on the microtiter plate is lower than the cut-off limit), green indicates sensitive strain while testing of antibiotic marked in gray is not required. LP: *Lpl. plantarum*; LB: *Lev. brevis*; LL: *L. lactis*; BC: *B. coagulans*. Cut-off MICs Non-EFSA antibiotics are not available therefore the strains cannot be classified as resistant or sensitive

The antibiotic susceptibility profiles of the bacterial strains were evaluated to assess their safety for probiotic and overall food/feed applications in accordance with EFSA guidelines. The antimicrobial susceptibility profiles of the bacterial strains were evaluated against antibiotics included in the EFSA panel as well as additional, non-EFSA antibiotics (Fig. 8). For EFSA-listed antibiotics, *Lpl. plantarum* and *Lev. brevis* displayed unclear susceptibility to kanamycin, streptomycin, and vancomycin due to concentration range limitations, whereas *B. coagulans* and *L. lactis* exhibited clear resistance to streptomycin and vancomycin. Resistance to tetracycline was observed in LB, LL, and BC, while *Lpl. plantarum* remained susceptible. Chloramphenicol, erythromycin and ampicillin susceptibility was consistent across all strains.

Overall, *Lpl. plantarum* exhibited the most favorable antimicrobial susceptibility profile, with the fewest confirmed resistances. *Lpl. plantarum* strains are generally susceptible to clindamycin; however, bimodal distribution of MICs suggests acquired resistance (Rozman et al. 2022). Notably, whole genome sequence analyses have not identified any known resistance genes or mutations that could account for this phenotype (Rozman et al. 2023). In contrast, *Lev. brevis* frequently exhibited multiple antibiotic resistances (Duche et al. 2023; Rozman et al. 2023; Singh et al. 2020), while resistance of *L. lactis* to antibiotics appeared to be strain dependent (Hamdaoui et al. 2024; Rozman et al. 2023). *B. coagulans* demonstrated the broadest resistance spectrum. Although antibiotic resistance is not frequently reported in *B. coagulans* strains (Altun and Erginkaya 2021; Bang et al. 2021; Kim, 2021), on the other hand, Sunhare et al. detected multiple resistance in a candidate *B. coagulans* strain for probiotics (Sunhare et al. 2024). These results highlight significant inter-strain variation in resistance patterns, which may have implications for strain selection in probiotic applications, particularly when safety assessments are guided by EFSA criteria. Interestingly, although *B. coagulans* ATCC 7050 is already recognized as a probiotic and included in commercial formulations (Mazzantini et al. 2022), its resistance to more than half of the EFSA-recommended antibiotics raises concerns regarding its suitability as a safe probiotic culture.

## Conclusion

Among the tested strains, the most promising probiotic candidates were *Lev. plantarum* and *K. marxianus* DSM 7238. For bacterial cultures, *Lpl. plantarum*, originating from halophyte biomass, demonstrated exceptional robustness. This was reflected in its high tolerance to NaCl and phenol, strong cell surface hydrophobicity, and good survivability under simulated digestion. It also exhibited superior biofilm formation, relatively high antioxidant activity, antimicrobial activity against two pathogens, moderate survival in simulated in vitro digestion, and the most favorable antibiotic susceptibility profile among the tested bacteria. These combined traits make *Lpl. plantarum* an attractive candidate for probiotic development and biotechnological applications.

Among the yeasts, *K. marxianus* DSM 7238 stood out as the most promising strain. Although all yeasts displayed high survivability after simulated digestion, strong autoaggregation, and high hydrophobicity, *K. marxianus* showed distinct advantages, mainly higher antioxidant activity, antimicrobial activity against three pathogens, and superior tolerance to NaCl and phenol stress. These characteristics highlight its potential not only as a probiotic but also as a valuable strain for biorefinery applications. In contrast, the established probiotic strain *B. coagulans* ATCC 7050 underperformed across most evaluated factors. It exhibited broad antibiotic resistance, raising potential safety concerns, as well as the lowest survivability under simulated digestion. Furthermore, it failed to grow in complex halophyte-based media, limiting its suitability for both probiotic use and biorefinery processes. A comparative summary of the key probiotic-related characteristics of the most promising strains identified in this study is provided in Table 5.

**Table 5:** Summary of probiotic-related properties of the most promising strains identified in this study

Probiotic property	Lpl. plantarum IM 2435	K. marxianus DSM 7238
NaCl tolerance	> 70% survivability at 5% NaCl	> 60% survivability at 5% NaCl
Phenol tolerance	> 80% survivability at 0.3% phenol	~ 80% survivability at 0.1% phenol
Simulated gastrointestinal survival	High survivability for bacterial culture (~ 30%) after *in vitro digestion*	High survivability (~ 100%) after in vitro digestion
Antimicrobial activity	Inhibition of *A. hydrophila* and *Cl. difficile*	Inhibition of *S. epidermidis* ATCC 12,228, *S. epidermidis* ATCC 14,990, *S. lugdunensis* M33-01
Antimicrobial mechanism	Acid-mediated (activity lost after neutralization)	Likely non-acid-dependent (yeast-associated mechanisms)
Autoaggregation	Moderate (> 40% after 24 h)	High (~ 90% after 24 h)
Cell surface hydrophobicity	High (~ 40–55%)	High (~ 55–70%)
Biofilm formation	High (A568 ≈ 4.3)	Low–moderate (A568 ≈ 0.8)
Antioxidant activity (DPPH scavenging)	Moderate (up to ~ 34% in supernatant)	Very high (up to ~ 74% in supernatant)
Antibiotic resistance profile	Resistance to clindamycin (CLI)	Not applicable (yeast)
Overall probiotic potential	Overall strong candidate for bacterial probiotic applications	Strong candidate for yeast-based probiotic and antioxidant applications

Together, these results demonstrate that halophyte-adapted strains such as *Lpl. plantarum* and *K. marxianus* DSM 7238 could provide a foundation for safe, resilient, and multifunctional probiotics suited to both human or animal health and industrial applications. To fully establish their safety profiles and probiotic potential, further whole genome sequencing and in vivo validation studies will be essential. Such investigations will also support the optimization of their use in functional foods and biotechnological processes.

## Data Availability

Data will be available on request to the corresponding author.
